# The Hypnotic Effect of Spinosin Is Mediated by Adenosine A_2A_ Receptors in Male Mice

**DOI:** 10.3390/nu18111785

**Published:** 2026-06-01

**Authors:** Jianping Zhang, Haimin Zhang, Wenrui Zhao, Lin Li, Lisheng Chu

**Affiliations:** 1 Department of Anatomy, Histology and Embryology, School of Basic Medical Sciences, Zhejiang Chinese Medical University, Hangzhou 310053, China; zhangjp@zcmu.edu.cn (J.Z.); zhm2410714450@163.com (H.Z.); zhaowenrui0917@163.com (W.Z.); 2 Department of Physiology, School of Basic Medical Sciences, Zhejiang Chinese Medical University, Hangzhou 310053, China; lilin@zcmu.edu.cn

**Keywords:** spinosin, caffeine, adenosine A_2A_ receptor, sleep, wakefulness

## Abstract

**Background/Objectives:** Insomnia is a prevalent clinical sleep disorder, with existing hypnotic therapies limited by safety concerns. There is an urgent clinical need for new safe, effective sleep-promoting candidates derived from natural products. Spinosin is one of the main active components of *Semen Ziziphi Spinosae* that exerts sedative and hypnotic effects. The adenosine receptor (AR) has been reported as a potential therapeutic target for insomnia; however, the hypnotic effect of spinosin through the A_2A_R remains to be elucidated. **Methods:** In the study, the involvement of A_2A_Rs in spinosin’s hypnotic effect was investigated using caffeine and further elucidated in A_2A_R-knockout (KO) mice. Diazepam was used as a positive control drug to validate the experimental model and evaluate the hypnotic effect of spinosin. Molecular docking and molecular dynamics (MDs) simulations were performed to validate the interaction of spinosin with the A_2A_R. **Results:** The hypnotic effects of spinosin were effectively antagonized by caffeine. Compared with A_2A_R-wild-type (WT) mice, spinosin-induced non-rapid eye movement (NREM) sleep and locomotor activity diminution were significantly reduced in A_2A_R-KO mice. Spinosin significantly increased the activity of γ-aminobutyric acid (GABA)ergic medium spiny neurons (MSNs) in the nucleus accumbens (NAc) and significantly decreased the activity of orexin neurons in the lateral hypothalamus (LH), as revealed by c-Fos immunostaining. These effects were significantly reversed by caffeine pretreatment or in A_2A_R-KO mice. Finally, the results of molecular docking showed that spinosin had a good binding potential with the A_2A_R. MD simulations further demonstrated that spinosin had strong binding stability with the A_2A_R. **Conclusions:** Our findings strongly suggest that spinosin exerts the hypnotic effects through the A_2A_R, and thus may have therapeutic potential for insomnia. Our identification of spinosin’s direct molecular target supports its translational potential as a novel natural-origin candidate for clinical insomnia drug development.

## 1. Introduction

Insomnia has increasingly become prevalent, primarily characterized by insufficient sleep duration and/or poor sleep quality and difficulty initiating or sustaining sleep [1]. Long-term insomnia contributes to emotional disturbances, daytime drowsiness, reduced work efficiency, and immune suppression, while increasing the risks of hypertension, neurasthenia, cardio-cerebrovascular events, and psychological disorders [2]. Traditional Chinese medicine (TCM) has gained increasing attention as a therapeutic approach for insomnia [3,4]. Among them, *Semen Ziziphi Spinosae* has long been used as a medicine-food homology substance in TCM for promoting sleep [5]. Spinosin, a natural flavonoid-C-glycoside (2″-β-O-glucopyranosyl flavone, C_28_H_32_O_15_) isolated from *Semen Ziziphi Spinosae* (Figure S1), has various biological activities [6,7], including antioxidant and anti-inflammatory activities, and possesses beneficial neuroprotective properties in neurological disorders such as Alzheimer’s disease [8] and anxiety [9]. Recently, spinosin has been reported to have hypnotic effects [10]. Modified Suanzaoren decoction, with spinosin as its primary active component, has been reported to reduce sleep onset latency and prolong sleep time in a mouse model of insomnia [11]. However, the effects of spinosin on sleep architecture require further elucidation, and its sleep-promoting mechanisms are not fully understood.

Adenosine, a key sleep regulatory substance in the brain, induces sleep by binding to its A_1_ and A_2A_ receptors (A_2A_Rs), with A_2A_Rs being more important [12,13]. However, the involvement of A_2A_R in the hypnotic effects mediated by spinosin remains uncertain.

Caffeine, a central nervous system stimulant in beverages such as coffee, tea, and energy drinks, is widely recognized for its sleep-disrupting effects [14,15]. Caffeine is also a nonspecific adenosine receptor antagonist, with similar affinity to both A_1_ and A_2A_R. While caffeine fails to disrupt sleep in mice with genetically abolished A_2A_R function, it promotes wakefulness in wild-type mice as well as transgenic mice lacking functional A_1_R [16]. We therefore hypothesize that spinosin may exert hypnotic effects via the A_2A_R. Currently, caffeine is widely used as a pharmacological tool to probe adenosine receptor function [17,18]. In the present study, caffeine more closely mimics real-life human exposure than selective A_2A_R antagonists do.

The regulation of sleep–wake states involves complex interactions across multiple brain regions and is governed by reciprocal inhibition between wake- and sleep-promoting neuronal populations via distinct neuromodulators and neurotransmitters [19]. Wake-promoting neurons include basal forebrain cholinergic neuron, monoaminergic cell groups, the orexin neuron of the lateral hypothalamus (LH), etc. [20]. Most sleep-promoting neuronal populations are γ-aminobutyric acid (GABA)ergic, including those in the ventrolateral preoptic nucleus (VLPO) [21], parafacial zone [22], and nucleus accumbens (NAc) [23]. Therefore, strategies designed to enhance the activity of sleep-promoting circuits or inhibit wake-active populations may offer novel therapeutic avenues for the development of hypnotic agents. In the NAc, A_2A_Rs are densely expressed on the GABAergic medium spiny neurons (MSNs) [24,25]. Hence, it is worth exploring whether spinosin affects the activity of NAc GABAergic MSNs and LH orexin neurons through the A_2A_R.

In the present study, the pentobarbital sodium-induced sleep test (PIST), the open field test (OFT), and electroencephalogram (EEG)/electromyogram (EMG) recordings were employed to confirm the antagonistic effect of caffeine on spinosin-induced hypnosis. Subsequently, the possible involvement of A_2A_R in spinosin-induced hypnosis was investigated using A_2A_R-knockout (KO) mice and their wild-type (WT) littermates. C-Fos immunostaining was employed to detect the effects of spinosin on the activity of sleep-promoting GABAergic MSNs in the NAc and wake-promoting orexin neurons in the LH. The binding interactions of spinosin with A_2A_R were validated using molecular docking and molecular dynamics (MD) techniques. Collectively, our findings provide insights into the molecular mechanisms underlying the hypnotic effects of spinosin, which may provide a potential therapeutic candidate for insomnia.

## 2. Materials and Methods

### 2.1. Animals

A total of 90 Male C57BL/6 mice aged 8–10 weeks and weighing between 22 and 26 g were purchased from Shanghai SLAC Laboratory Animal Co., Ltd. (Shanghai, China). A total of 25 A_2A_R-KO mice and their 30 WT littermates (8–10 weeks and weighing between 22 and 25 g) of the inbred C57BL/6 strain were generated from heterozygotes kindly provided by Jiang-fan Chen (Wenzhou Medical University) [26]. All mice were housed in the Laboratory Animal Research Center of Zhejiang Chinese Medical University under controlled conditions (23 ± 2 °C, 40–60% humidity) with a 12 h light/dark cycle (lights on at 7:00, off at 19:00). Experiments were initiated after one week of acclimatization with ad libitum access to food and water. After completing all behavioral tests in this study, mice were euthanized by gradual carbon dioxide (CO_2_) inhalation followed by cervical dislocation as a secondary confirmation method. The animal study was performed with the approval of the Institutional Animal Care and Use Committee of Zhejiang Chinese Medical University (approval NO. IACUC-20220627-07).

### 2.2. Grouping and Pharmacological Treatments

Mice were randomly assigned to five groups: the control group, the spinosin (40 mg/kg) group, the caffeine (5 mg/kg) group, the caffeine (5 mg/kg) combined with spinosin (40 mg/kg) group, and the caffeine (5 mg/kg) combined with diazepam (15 mg/kg) group, with 5 mice in each group. A_2A_R-KO mice and their A_2A_R-WT littermates were randomly assigned to two groups, the control group and the spinosin (40 mg/kg) group, with 5 mice in each group. The mice, individually numbered from 1 to N, were randomly allocated to experimental groups using a random number table. Spinosin (purity: 94.8%, Lot No. 111869-202005) was dissolved in a vehicle consisting of 1.6% Tween-20, 4.9% DMSO, 10% (*v*/*v*) 95% ethanol and normal saline, as it has poor aqueous solubility and low lipophilicity. Diazepam, used as the positive control, was purchased from the Tianjin Pharmaceutical Peace Co., Ltd. (Batch No. 2503291, Tianjin, China) and dissolved in normal saline. Caffeine (Sigma-Aldrich, St. Louis, MO, USA, NO. C0750), purchased from a licensed supplier and used in accordance with local regulations, was dissolved in normal saline. Spinosin, diazepam, and vehicle were administered via intraperitoneal (i.p.) injection at a dose of 10 mL/kg body weight at 21:00 on the day of the experiment. Caffeine was injected i.p. 5 min prior to the administration of spinosin, vehicle, or diazepam. This study was conducted from September 2022 to September 2025, with a total study duration of 3 years.

### 2.3. Pentobarbital Sodium-Induced Sleep Test (PIST)

The tests were performed in accordance with a previously reported method by Gong et al. [27] with some modifications, in which pentobarbital sodium (45 mg/kg, i.p.) was used as the threshold dose. Mice received the spinosin, diazepam, or vehicle 1 h before the administration of pentobarbital sodium (i.p.). The pentobarbital-induced loss of righting reflex (LORR) was confirmed if the mice remained in dorsal recumbency for 10 s [28]. The sleep onset latency (time from pentobarbitone administration to LORR) and sleep duration (time from LORR to recovery of righting reflex) were recorded in each group [29].

### 2.4. EMG Recording Electrode Implantations

Procedures for EEG/EMG recording electrodes implantations were adapted from the previously described protocol [30]. To monitor EEG signals, two stainless steel screws were inserted into the skull (AP, +1.0 mm; LR, −1.5 mm from bregma). For EMG implantation, two Teflon-coated stainless-steel wires were bilaterally placed into both trapezius muscles. All the electrodes were attached to a mini-connector and fixed to the skull with dental cement. Mice were housed individually for 7 days to allow recovery.

### 2.5. Recording and Analysis

Mice were connected to a cable and allowed to habituate to the recording chamber. EEG/EMG signals were recorded using the Medusa Tethered Acquisition System (Medusa, Bio-Signal Technologies, Nanjing, China) at a sampling rate of 1000 Hz. The signals were captured, amplified, and subjected to selective frequency filtering (EEG, 0.5–70 Hz; EMG, 30–90 Hz). EEG/EMG signals were recorded continuously from 19:00 to 00:00. Drugs were administered intraperitoneally at 21:00. Data were analyzed using Lunion Stage automatic sleep scoring software (Version number: 1.1.20220308.1910, Lunion Data, Shanghai, China) with 4 s as a unit. The relevant parameters were set on the software for automatic analysis of brainwaves. Sleep recordings were automatically scored in 4 s epochs into three stages [wake, non-rapid eye movement sleep (NREM; NREM sleep is one of the two main phases of human sleep characterized by the absence of rapid eye movement during sleep, a decrease in brain activity, and a general slowdown in bodily functions), and rapid eye movement (REM; REM sleep is a specific sleep stage within the sleep cycle and, together with NREM sleep, forms the two fundamental stages of the human sleep cycle)] by the AI-driven software Lunion Stage, followed by manual verification. The wake stage was characterized by low-amplitude and high-frequency desynchronized EEG with high EMG activity. The NREM sleep is characterized by a synchronized EEG with low-frequency (0.5–4 Hz), high-amplitude delta waves with low-amplitude EMG waves. The REM sleep is characterized by prominent bands in the theta frequency (4–8 Hz) of the EEG and low-amplitude EMG waves. All brainwaves were derived and analyzed after manual correction for statistical analysis and mapping.

### 2.6. Open Field Test (OFT)

The OFT is a classic neurobehavioral experimental method used to study the spontaneous activities, exploration behaviors, and anxiety levels of rodents. The OFT was conducted one hour after treatment in mice, as previously described [31]. The mice were placed in the center of an OFT box (40 × 40 × 40 cm), which was divided into 4 squares of equal area. The movement of each mouse in the square was recorded by camera for a duration of 10 min. The data were analyzed using the Any-maze animal behavior analysis software (Version number: 6.x, Stoelting Co., Ltd., Wood Dale, IL, USA), and the total distance moved and the average speed were recorded.

### 2.7. Immunofluorescence Staining

One hour after the administration of spinosin or saline, mice were anesthetized with pentobarbital (60 mg/kg, i.p.) and transcardially perfused with saline followed by 4% paraformaldehyde (PFA). The specimens were post-fixed in 4% PFA overnight at 4 °C. Following fixation, the brain tissues were subjected to dehydration through the graded sucrose solutions (20% and 30%) at 4 °C. Using a freezing microtome (Thermo Fisher Scientific, Waltham, MA, USA), 30 μm thick coronal sections were cut and stored in a cryoprotectant solution for subsequent staining.

The sections were blocked for nonspecific binding of the secondary antibody in a solution containing 10% normal goat serum (NGS) and 0.3% Triton X-100 in phosphate-buffered saline (PBS) for 1 h. For double immunofluorescence staining of c-Fos/cAMP-regulated phosphoprotein-32 (DARPP-32, a marker for GABAergic MSNs [32,33]) in the NAc or c-Fos/orexin-A in the LH, brain sections were incubated in a mixture of monoclonal rabbit anti-c-Fos antiserum (Abcam, Cambridge, UK; Catalogue No. ab214672; 1:1000) and monoclonal mouse anti-DARPP-32 antiserum (Santa Cruz Biotechnology, Inc., Dallas, TX, USA; Catalogue No. sc-135877; 1:400) or monoclonal mouse anti-orexin-A antiserum (Santa Cruz Biotechnology, Inc., USA; Catalogue No. sc-80263; 1:500) in PBS containing 5% NGS over night at 4 °C. After thorough rinsing in PBS, the sections were incubated in a mixture of Alexa Fluor 488-conjugated goat anti-rabbit IgG (Invitrogen, Carlsbad, CA, USA; Catalogue No. A11034; 1:1000) and Alexa Fluor 594-conjugated goat anti-mouse IgG (Invitrogen, USA; Catalogue No. A11005; 1:1000) for 2 h in darkness at room temperature. The sections were then washed and stained with DAPI (Solarbio, Beijing, China; Catalogue No. C0065), followed by final PBS washes and slide mounting for fluorescence microscopy (Olympus, Tokyo, Japan, model VS120-S6-W, instrument ID: 2017300706).

### 2.8. Molecular Docking Process

As previously described [34], the homology model of ADORA2A (A_2A_R) for molecular docking was used in the present study. The 3D structure of the ADORA2A (PDB ID: 5NM4) was downloaded from the Protein Data Bank (PDB) database (http://www.rcsb.org/pdb/home/home.do, accessed on 26 September 2025) and processed with PyMOL 2.6.0 to remove water molecules and phosphate ions. The resulting structure was saved as PDB file. The 2D structures of spinosin, caffeine and adenosine were obtained from the PubChem database (http://pubchem.ncbi.nlm.nih.gov/). These 2D structures were imported into ChemOffice 20.0 to generate their 3D structures, which were exported as mol2 files. Energy minimization of ligands (spinosin, caffeine, and adenosine), as well as preprocessing such as the identification of active pockets of the target protein A_2A_R, was performed using the Molecular Operating Environment (MOE) 2019 software. Molecular docking was carried out using MOE 2019, with 50 runs performed per ligand. The binding affinities were evaluated based on the calculated binding energies. The docking results were visualized using PyMOL 2.6.0 and Discovery Studio 2019 software.

### 2.9. Molecular Dynamics (MD) Simulations

MD simulations were conducted using the GROMACS 2020 package with the AMBER99SB-ILDN force field and TIP3P explicit water model, and the system was electrically neutral by adding counterions. Subsequently, energy minimization (1000.0 kJ/mol/nm) was performed using the steepest descent method. Canonical ensemble simulation (NVT, 400 ps) and isothermal isobaric simulation (NPT, 400 ps) were used to guarantee that the system could endure constant temperature and pressure (300 K, 1 bar). The annealing method was used to ensure that the system was slowly warmed from 0 to 300 K within 50 ps in the NVT simulation process. Finally, a 100 ns MD simulation was started. The Gbinding energy of ligands and proteins was calculated using the gmx MMPBSA method of the Gromacs 2020 program.

### 2.10. Statistical Analysis

All data were presented as mean ± standard error of the mean (SEM). All data were first tested for normality by Shapiro–Wilk test. Normally distributed data were analyzed using parametric tests, such as two-way ANOVA followed by Sidak’s multiple comparisons test or unpaired t-tests. Non-normally distributed data were analyzed using nonparametric tests, such as the Kruskal–Wallis test followed by Dunn’s multiple comparisons test (for ≥3 groups) or the Mann–Whitney U test (for exactly 2 groups). Neuron counts were quantified using ImageJ (Version number: 1.54 n) in a blinded fashion. Statistical analysis was performed by GraphPad Prism 8.0 (GraphPad Software, San Diego, CA, USA), with statistical significance set at *p* < 0.05. To eliminate observer bias, all experiments were strictly blinded. Unblinding occurred only after all data analyses were completed.

## 3. Results

### 3.1. Spinosin-Induced Hypnotic Effects Were Antagonized by Caffeine Pretreatment in Mice

**Figure 1:**
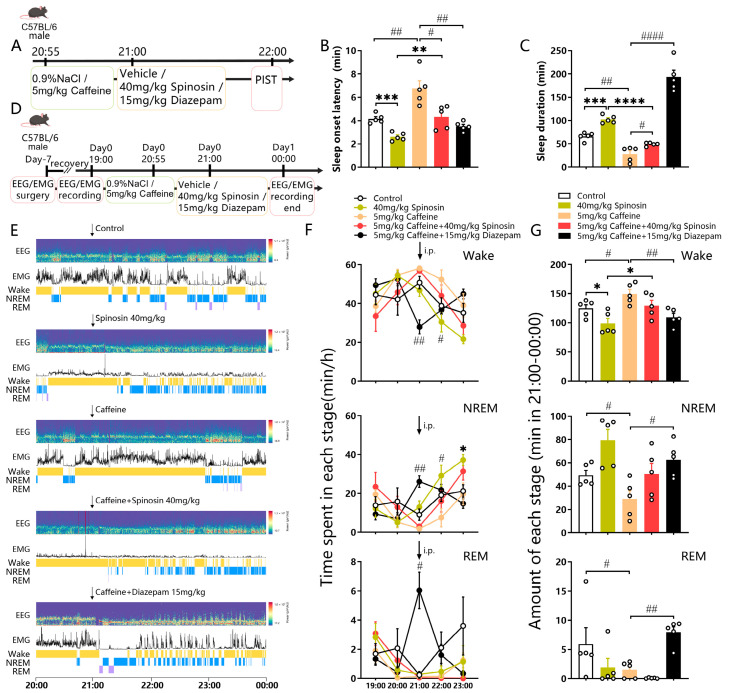
Hypnotic effects of spinosin are antagonized by caffeine pretreatment. (**A**) The pentobarbital sodium-induced sleep test (PIST) study scheme: spinosin (40 mg/kg, i.p.) and caffeine (5 mg/kg, i.p.) were administered 60 min and 65 min before pentobarbital sodium, respectively. (**B**) Changes in sleep onset latency in mice in the PIST study. (**C**) Changes in sleep duration in mice in the PIST study. (**D**) The experimental scheme of the electroencephalogram (EEG)/electromyogram (EMG) recordings: EEG and EMG recordings were collected from 19:00 to 00:00 on the testing day (5 h total). Spinosin (40 mg/kg, i.p.) was administered at 21:00, and caffeine (5 mg/kg, i.p.) was administered 5 min prior. (**E**) Typical examples of polygraphic recordings and corresponding hypnograms illustrating changes in sleep over 4 h (20:00–00:00) following vehicle or drug administration. Arrows indicate the time of injection. (**F**) Time course of wakefulness, non-rapid eye movement (NREM) sleep, and rapid eye movement (REM) sleep in mice following vehicle or drug administration. Arrows indicate the time of injection. (**G**) Total duration of wakefulness, NREM sleep, and REM sleep for 3 h in mice following vehicle or drug administration. Data are presented as mean ± SEM (*n* = 5/group). * *p* < 0.05, ** *p* < 0.01, *** *p* < 0.001, **** *p* < 0.0001, ^#^ *p* < 0.05, ^##^ *p* < 0.01, ^####^ *p* < 0.0001.

To examine whether caffeine significantly antagonizes the hypnotic effects of spinosin, we performed the PIST, EEG/EMG recordings, and OFT in mice. We previously reported that the hypnotic effect of spinosin at 40 mg/kg appeared to be similar to that of the positive control (20 mg/kg of diazepam) in mice [35]. Therefore, a spinosin concentration of 40 mg/kg was used in all subsequent experiments. It was found that, consistent with the previous report [36], spinosin significantly reduced sleep-onset latency by 37.02% (from 4.16 ± 0.18 min to 2.62 ± 0.16 min, *p* = 0.0002) and extended sleep duration by 55.22% (from 65.99 ± 3.91 min to 102.43 ± 3.72 min, *p* = 0.0001) in the PIST (Figure 1B,C). At a dose of 5mg/kg, caffeine significantly prolonged sleep-onset latency by 62.74% (from 4.16 ± 0.18 min to 6.77 ± 0.64 min, *p* = 0.0045) and shortened sleep duration by 57.18% (from 65.99 ± 3.91 min to 28.26 ± 7.10 min, *p* = 0.0016) (Figure 1B,C). Furthermore, pretreatment with caffeine antagonized the hypnotic effects of spinosin (Figure 1B,C). Representative examples of polysomnographic recordings and corresponding hypnograms illustrating changes in sleep over 4 h (20:00–00:00) were presented in Figure 1E. Total time spent in wakefulness, NREM sleep, and REM sleep was measured for 3 h following the administration of spinosin or caffeine (Figure 1G). Our results, consistent with the previous reports [35], revealed that spinosin induced a significant reduction in the duration of wakefulness by 20.76% (from 124.69 ± 6.30 min to 98.81 ± 8.53 min, *p* = 0.0405) and a slight increase in NREM sleep duration by 60.64% (from 49.36 ± 4.18 min to 79.29 ± 9.37 min, *p* = 0.0760) when compared to the control group. In contrast, caffeine treatment significantly increased the duration of wakefulness by 19.86% (from 124.69 ± 6.30 min to 149.45 ± 7.74 min, *p* = 0.0380) and significantly reduced NREM sleep duration by 41.23% (from 49.36 ± 4.18 min to 29.01 ± 7.15 min, *p* = 0.0470). Additionally, compared to the spinosin group, pretreatment with caffeine exhibited a significant increase in the duration of wakefulness by 31.00% (from 98.81 ± 8.53 min to 129.44 ± 9.10 min, *p* = 0.0395) and a slight reduction in NREM sleep duration by 36.33% (from 79.29 ± 9.37 min to 50.48 ± 9.07 min, *p* = 0.0760).

**Figure 2:**
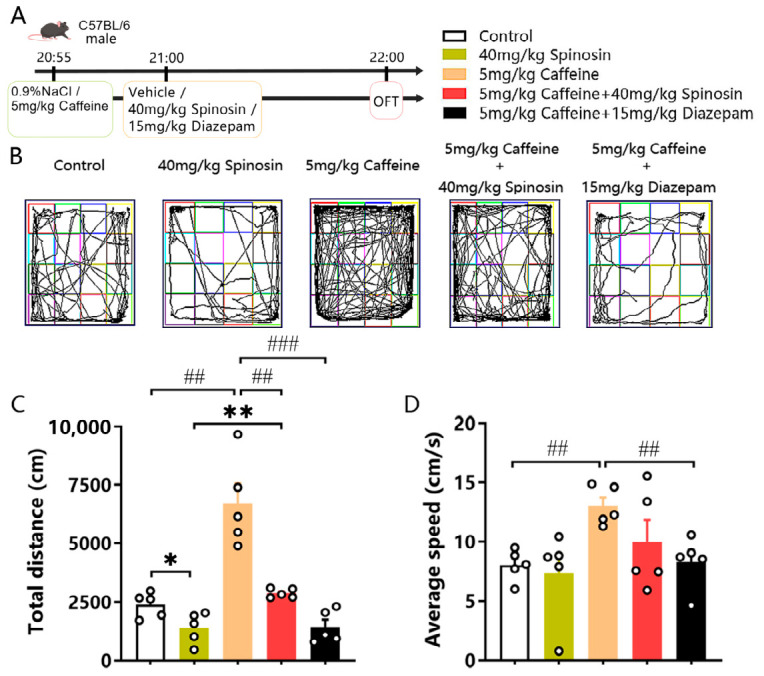
Sedative effects of spinosin are antagonized by caffeine pretreament in the open field test (OFT). (**A**) The OFT scheme: spinosin (40 mg/kg, i.p.) and caffeine (5 mg/kg, i.p.) were administered 60 min and 65 min before pentobarbital sodium, respectively. (**B**) Representative trajectories illustrating changes in spontaneous activity in OFT in mice following vehicle or drug administration. (**C**) Changes in total distance in mice in the OFT following vehicle or drug administration. (**D**) Changes in average speed in mice in the OFT following vehicle or drug administration. Data are presented as mean ± SEM (*n* = 5/group). * *p* < 0.05, ** *p* < 0.01, ^##^ *p* < 0.01, ^###^ *p* < 0.001.

In the OFT findings (Figure 2B–D), spinosin-treated mice traveled a significantly shorter distance than control mice by 41.37% (from 2384.57 ± 239.33 cm to 1398.19 ± 293.32 cm, *p* = 0.0313) (Figure 2C). Consistent with the previous report [15], caffeine-treated mice exhibited significantly increased hyperactivity, as evidenced by a rise in total distance traveled by 181.52% (from 2384.57 ± 239.33 cm to 6713.13 ± 845.25 cm, *p* = 0.0012) and in average speed by 61.69% (from 8.04 ± 0.59 cm/s to 13.00 ± 0.73 cm/s, *p* = 0.0090) (Figure 2C,D). Additionally, compared to the spinosin group, caffeine pretreatment significantly increased total distance by 105.71% (from 1398.19 ± 293.32 cm to 2876.28 ± 87.38 cm, *p* = 0.0013) (Figure 2C), suggesting the sedative effect induced by spinosin may be related to the A_2A_R. Collectively, these findings from the PIST, EEG/EMG recordings, and OFT suggest that the hypnotic effects of spinosin are mediated by the A_2A_R.

### 3.2. Effect of Caffeine Pretreatment on Spinosin-Induced Changes in Sleep Architecture in Mice

**Figure 3:**
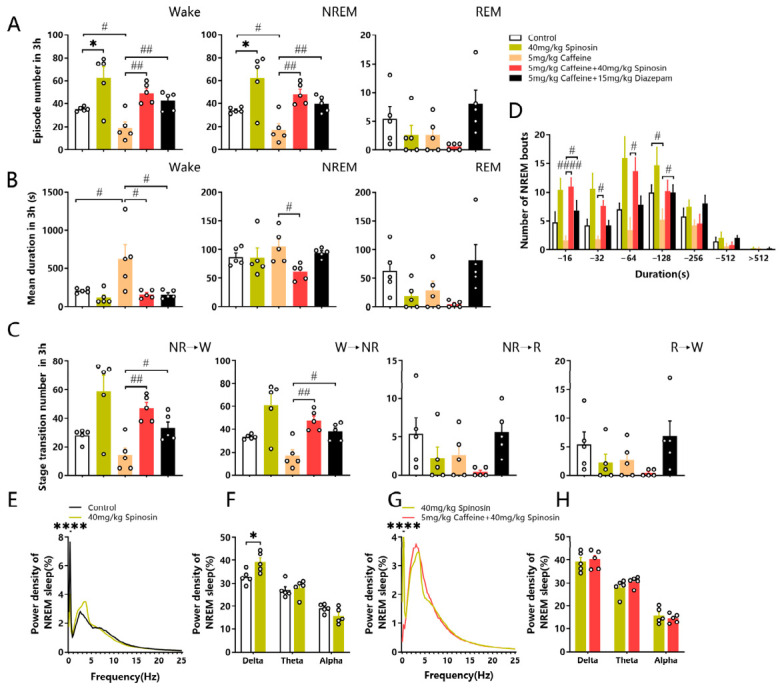
Effects of caffeine pretreatment on spinosin-induced changes in sleep architecture and electroencephalogram (EEG) power spectrum of non-rapid eye movement (NREM) sleep obtained from the EEG/electromyogram (EMG) recordings during 21:00–00:00. (**A**) Episode number of wakefulness, NREM sleep, and rapid eye movement (REM) sleep in mice following vehicle or drug administration. (**B**) Mean duration of wakefulness, NREM sleep, and REM sleep in mice following vehicle or drug administration. (**C**) Transitions between W (wakefulness), NR (NREM sleep), and R (REM sleep) in mice following vehicle or drug administration. (**D**) The number of NREM sleep bouts with different durations in mice following vehicle or drug administration. (**E**) EEG power density curves of NREM sleep after administration of vehicle or spinosin (40 mg/kg) in mice. (**F**) EEG power density of Delta, Theta, and Alpha bands during NREM sleep after administration of vehicle or spinosin (40 mg/kg) in mice. (**G**) EEG power density curves of NREM sleep after administration of spinosin (40 mg/kg) or caffeine (5 mg/kg) with spinosin (40 mg/kg). (**H**) EEG power density of delta, theta, and alpha bands during NREM sleep in mice following administration of spinosin (40 mg/kg) alone or in combination with caffeine (5 mg/kg). Data are presented as mean ± SEM (*n* = 5/group). * *p* < 0.05, **** *p* < 0.0001, ^#^ *p* < 0.05, ^##^ *p* < 0.01, ^####^ *p* < 0.0001. Horizontal bars indicate location of a statistically significant difference (**E**,**G**).

To better understand the hypnotic profile of spinosin, we analyzed the episode number and mean duration of each sleep–wake stage, as well as transitions between wakefulness, NREM sleep, and REM sleep. Consistent with our previous reports [35], spinosin significantly increased the number of wakefulness episodes by 77.27% (from 35.20 ± 0.86 episodes to 62.40 ± 10.03 episodes, *p* = 0.0270) and NREM sleep episodes by 82.35% (from 34.00 ± 1.10 episodes to 62.00 ± 10.35 episodes, *p* = 0.0275) (Figure 3A), while it had a slight increase in the mean duration of NREM sleep (Figure 3B), the transition frequency between wakefulness and NREM sleep) (Figure 3C), and the number of NREM sleep bouts (Figure 3D). However, pretreatment with caffeine did not significantly antagonized spinosin’s effect on these sleep parameters (Figure 3A–D), suggesting that the effects of spinosin on sleep architecture could not be completely antagonized by caffeine.

### 3.3. Effect of Caffeine Pretreatment on Spinosin-Induced Changes in EEG Delta Power Spectrum of NREM Sleep in Mice

To further explore the hypnotic effect of spinosin, we analyzed its effects on EEG delta power spectrum during NREM sleep from 21:00 to 00:00. The power in each 0.5 Hz bin is first averaged within each sleep stage and then normalized by calculating the relative power of each bin with respect to the total power (0–25 Hz) for each individual mouse. Compared to the control group, spinosin significantly increased the delta power density by 6.46% (from 32.73 ± 1.31% to 39.19 ± 1.93%, *p* = 0.0172) during NREM sleep in mice (Figure 3E,F). With caffeine pretreatment, spinosin still significantly increased delta power density during NREM sleep (Figure 3G,H). These results indicate that the increased delta power density induced by spinosin could not be completely antagonized by caffeine.

### 3.4. The Hypnotic Effects of Spinosin Were Significantly Reduced in A_2A_R-KO Mice

**Figure 4:**
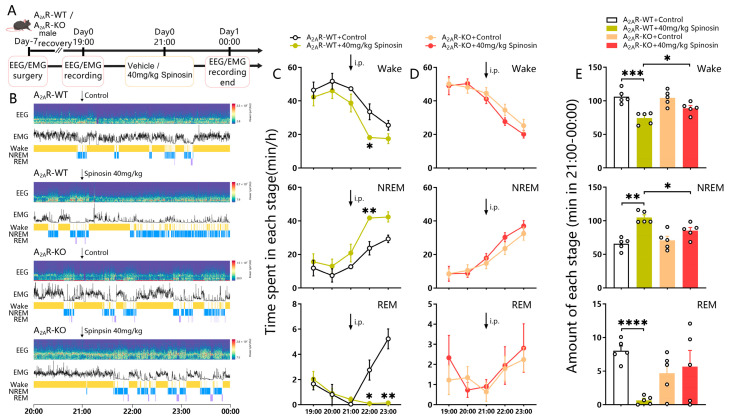
Hypnotic effects of spinosin are significantly reduced in adenosine A_2A_ receptor (A_2A_R)-knockout (KO) mice. (**A**) The experimental scheme of the electroencephalogram (EEG)/electromyogram (EMG) recordings: EEG and EMG recordings were collected from 19:00 to 00:00 on the testing day (5 h total) in A_2A_R-KO mice and their A_2A_R-WT littermates. Spinosin (40 mg/kg, i.p.) was administered at 21:00. (**B**) Typical examples of polygraphic recordings and corresponding hypnograms illustrating changes in sleep over 4 h (20:00–00:00) in A_2A_R-KO mice and their A_2A_R-wild-type (WT) littermates following vehicle or spinosin (40 mg/kg) administration. Arrows indicate the time of injection. (**C**,**D**) Time course of wakefulness, non-rapid eye movement (NREM) sleep, and rapid eye movement (REM) sleep in A_2A_R-KO mice (**D**) and their A_2A_R-WT littermates (**C**) following vehicle or spinosin (40 mg/kg) administration. Arrows indicate the time of injection. (**E**) Total duration of wakefulness, NREM sleep, and REM sleep for 3 h (21:00–00:00) in A_2A_R-KO mice and their A_2A_R-WT littermates following vehicle or spinosin (40 mg/kg) administration. Data are presented as mean ± SEM (*n* = 5/group). * *p* < 0.05, ** *p* < 0.01, *** *p* < 0.001, **** *p* < 0.0001.

To further elucidate the key role of A_2A_R in the hypnotic effects of spinosin, the present study employed A_2A_R-KO mice and their WT littermates in the EEG/EMG recordings. Representative examples of polysomnographic recordings and corresponding hypnograms illustrating changes in sleep over 4 h (20:00–00:00) were presented in Figure 4B. Compared with their own control, spinosin significantly reduced the duration of wakefulness by 45.91% (from 33.52 ± 4.70 min to 18.13 ± 1.21 min, *p* = 0.0492) and significantly increased the duration of NREM sleep by 76.10% (from 23.72 ± 3.97 min to 41.77 ± 1.22 min, *p* = 0.0070) in A_2A_R-WT mice at 22:00 (Figure 4C). Analysis of the total duration of wakefulness, NREM sleep, and REM sleep from 21:00 to 00:00 revealed that compared with their own control, spinosin significantly decreased the duration of wakefulness by 30.09% (from 106.29 ± 4.67 min to 74.31 ± 3.97 min, *p* = 0.0008) and significantly increased NREM sleep duration by 59.93% (from 65.68 ± 4.06 min to 105.04 ± 3.98 min, *p* = 0.0090) in A_2A_R-WT mice (Figure 4E). However, A_2A_R-KO mice treated with spinosin did not exhibit a significant increase in the duration of NREM sleep compared to their own control (Figure 4D,E). Compared to A_2A_R WT mice, A_2A_R-KO mice treated with spinosin exhibited a significant decrease in NREM sleep by 18.80% (from 105.04 ± 3.98 min to 85.29 ± 4.95 min, *p* = 0.0210) and a significant increase in wakefulness by 19.82% (from 74.31 ± 3.97 min to 89.04 ± 3.90 min, *p* = 0.0293) (Figure 4E).

**Figure 5:**
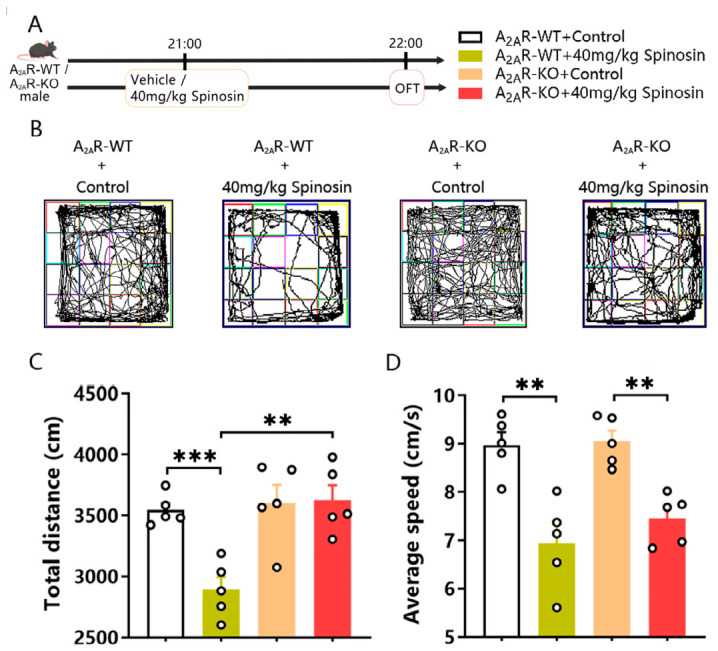
Sedative effects of spinosin are significantly reduced in adenosine A_2A_ receptor (A_2A_R)-knockout (KO) mice in the open field test (OFT). (**A**) The OFT scheme: spinosin (40 mg/kg, i.p.) was administered 60 min before the OFT in A_2A_R-KO mice and their A_2A_R- wild-type (WT) littermates. (**B**) Representative trajectories illustrating changes in spontaneous activity in OFT in A_2A_R-KO mice and their A_2A_R-WT littermates following vehicle or spinosin (40 mg/kg) administration. (**C**) Changes in total distance in A_2A_R-KO mice and their A_2A_R-WT littermates in the OFT following vehicle or spinosin (40 mg/kg) administration. (**D**) Changes in average speed in A_2A_R-KO mice and their A_2A_R-WT littermates in the OFT following vehicle or spinosin (40 mg/kg) administration. Data are presented as mean ± SEM (*n* = 5/group). ** *p* < 0.01, *** *p* < 0.001.

The OFT findings revealed that compared to their own control, spinosin significantly decreased total distance by 18.35% (from 3544.29 ± 56.35 cm to 2893.80 ± 102.38 cm, *p* = 0.0005) and average speed by 22.63% (from 8.97 ± 0.27 cm/s to 6.94 ± 0.41 cm/s, *p* = 0.0031) in A_2A_R-WT mice (Figure 5C,D). However, A_2A_R-KO mice treated with spinosin did not exhibit a significant reduction in total distance compared to their own control (Figure 5C). Additionally, compared to A_2A_R WT mice, A_2A_R-KO mice treated with spinosin exhibited a significant increase in total distance by 25.24% (from 2893.80 ± 102.38 cm to 3624.28 ± 122.90 cm, *p* = 0.0018) (Figure 5C). Collectively, these findings strongly suggest that A_2A_R mediates the hypnotic effects of spinosin.

### 3.5. Spinosin’s Effects on the Activity of NAc GABAergic MSNs and LH Orexin Neurons Were Significantly Reversed in Caffeine-Pretreated or A_2A_R-KO Mice

**Figure 6:**
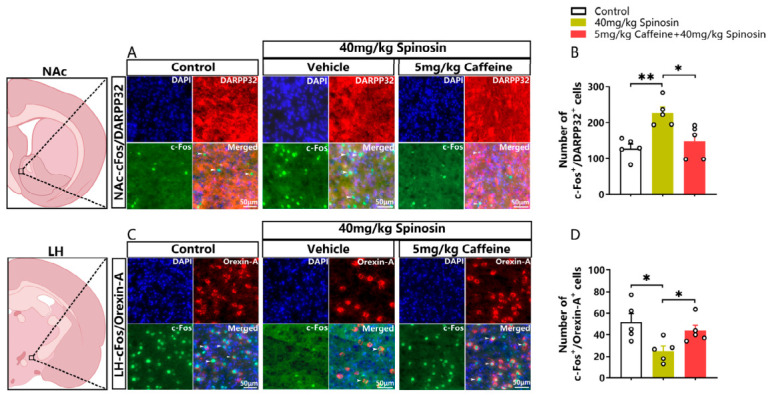
Effects of spinosin on the activity of γ-aminobutyric acid (GABA)ergic medium spiny neurons (MSNs) in the nucleus accumbens (NAc) and orexin neurons in the lateral hypothalamus (LH) are antagonized by caffeine pretreatment. (**A**) Representative immunofluorescence images showing co-expression of DAPI- (blue), c-Fos- (green), and cAMP-regulated phosphoprotein 32 (DARPP-32) (red) in the NAc. (**B**) Quantitative analysis of co-expression of c-Fos and DARPP-32 in the NAc. (**C**) Representative images showing co-expression of DAPI (blue), c-Fos (green), and orexin-A (red) in the LH. (**D**) Quantitative analysis of co-expression of c-Fos and orexin-A in the LH. Co-expression of DAPI (blue), c-Fos (green), and DARPP-32/orexin-A (red) is shown in the bottom right corner of each panel. White arrows indicate c-Fos-positive cells. Data are presented as mean ± SEM (*n* = 5/group). * *p* < 0.05, ** *p* < 0.01. Scale bar = 50 μm.

**Figure 7:**
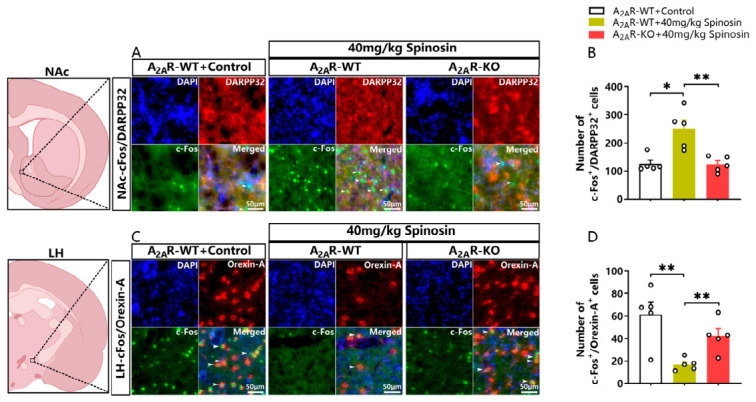
Effects of spinosin on the activity of γ-aminobutyric acid (GABA)ergic medium spiny neurons (MSNs) in the nucleus accumbens (NAc) and orexin neurons in the lateral hypothalamus (LH) are significantly reduced in adenosine A_2A_ receptor (A_2A_R)-knockout (KO) mice. (**A**) Representative images showing co-expression of DAPI- (blue), c-Fos- (green), and cAMP-regulated phosphoprotein 32 (DARPP-32) (red) in the NAc. (**B**) Quantitative analysis of co-expression of c-Fos and DARPP-32 in the NAc. (**C**) Representative images showing co-expression of DAPI (blue), c-Fos (green), and orexin-A (red). (**D**) Quantitative analysis of expression of c-Fos and orexin-A in the LH. Co-expression of DAPI (blue), c-Fos (green), and DARPP-32/orexin-A (red) is shown in the bottom right corner of each panel. White arrows indicate c-Fos positive cells. Data are presented as mean ± SEM (*n* = 5/group). * *p* < 0.05, ** *p* < 0.01. Scale bar = 50 μm.

To investigate whether the hypnotic effects of spinosin are related to sleep–wake-regulating neurons, we performed immunofluorescence staining of c-Fos, a well-established marker of neuronal activation [37], to evaluate the activity of NAc GABAergic MSNs and LH orexin neurons. In line with our prior findings [35], spinosin enhanced c-Fos and DARPP-32 co-expression in the NAc (from 127.56 ± 12.75 positive cells to 226.20 ± 16.37 positive cells, *p* = 0.0014, Figure 6) and (from 125.32 ± 12.76 positive cells to 250.56 ± 30.86 positive cells, *p* = 0.0160, Figure 7) and reduced c-Fos and orexin-A co-expression in the LH (from 51.84 ± 7.87 positive cells to 25.04 ± 4.60 positive cells, *p* = 0.0187, Figure 6) and (from 61.36 ± 10.95 positive cells to 16.92 ± 2.41 positive cells, *p* = 0.0042, Figure 7) compared to control mice. However, caffeine pretreatment significantly antagonized spinosin’s effects by reducing the co-expression of c-Fos and DARPP-32 in the NAc by 34.95% (from 226.20 ± 16.37 positive cells to 147.14 ± 20.49 positive cells, *p* = 0.0167) and concurrently increasing the co-expression of c-Fos and orexin-A in the LH by 74.16% (from 25.04 ± 4.60 positive cells to 43.61 ± 5.24 positive cells, *p* = 0.0287) (Figure 6). Furthermore, the effects of spinosin were significantly reduced in A_2A_R-KO mice, with a reduction in co-expression of c-Fos and DARPP-32 in the NAc by 50.75% (from 250.56 ± 30.86 positive cells to 123.40 ± 12.49 positive cells, *p* = 0.0090) and a concurrent increase in co-expression of c-Fos and orexin-A in the LH by 150.65% (from 16.92 ± 2.41 positive cells to 42.41 ± 6.44 positive cells, *p* = 0.0060) (Figure 7). These findings suggest that the hypnotic effects of spinosin may be related to the A_2A_R-mediated regulation of the activity of NAc GABAergic MSNs and LH orexin neurons.

### 3.6. Molecular Docking of Spinosin with ADORA2A

To examine the binding mode and affinity of spinosin for ADORA2A, we conducted molecular docking of spinosin, caffeine, and adenosine against ADORA2A. The binding energies between spinosin and ADORA2A, caffeine and ADORA2A, and adenosine and ADORA2A were −7.9666 kcal/mol, −6.0082 kcal/mol, and −6.6236 kcal/mol, respectively, indicating that the binding energy between spinosin and ADORA2A is the strongest among the three compounds.

**Figure 8:**
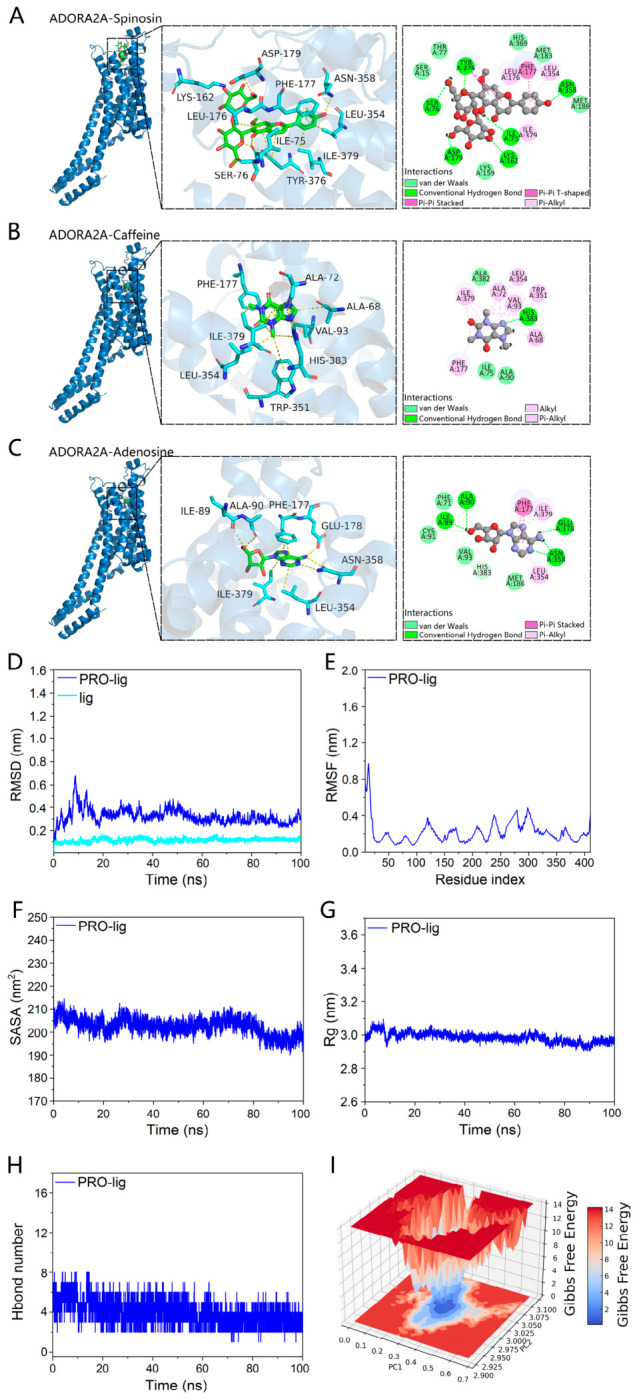
Molecular docking and molecular dynamics (MD) simulations of spinosin binding to ADORA2A. Predicted binding conformations and key interactions between ADORA2A and three compounds: (**A**) Spinosin. (**B**) Caffeine. (**C**) Adenosine. MD simulation analyses of spinosin-ADORA2A complexes at four different binding residues: (**D**) Values of root mean square deviation (RMSD). (**E**) Root mean square fluctuation (RMSF) curves. (**F**) Solvent-accessible surface area (SASA) curves. (**G**) Radius of gyration (Rg) curves. (**H**) Hydrogen bonds change curves. (**I**) Energy trap analysis results.

Spinosin formed six hydrogen bonds with ADORA2A residues including Ile75, Ser76, Lys162, Asp179, Asn358, and Tyr376 and hydrophobic interactions with ADORA2A residues including Leu176, Leu354, Ile379, Phe177, and Tyr376 (Figure 8A). Caffeine formed hydrogen bonds with ADORA2A residue His383 and hydrophobic interactions with ADORA2A residues including Ala68, Ala72, Val93, Phe177, Trp351, Leu354, and Ile379 (Figure 8B). Adenosine formed hydrogen bonds with ADORA2A residues including Ile89, Ala90, Glu178, Asn358 and hydrophobic interactions with ADORA2A residues including Phe177, Ile379, and Leu354 (Figure 8C). Compared to caffeine and adenosine, spinosin formed more hydrogen bonds with ADORA2A, suggesting significantly stronger binding affinity (Figure 8A).

### 3.7. MD Simulations

Spinosin docked to ADORA2A with the lowest binding energy, showing a binding affinity of −7.9666 kcal/mol, which prompted MD simulations to assess complex stability. The root mean square deviation (RMSD) value of the spinosyn–ADORA2A complex reached equilibrium at approximately 60 ns, with the mean RMSD below 0.5 nm, indicating that spinosin forms a stable complex with ADORA2A following equilibration (Figure 8D). Root mean square fluctuation (RMSF) analysis revealed that certain amino acid residues near the N-terminus and residue 300 exhibited relatively high conformational flexibility. These regions were located within the hinge region of the protein, which was inherently dynamic, thus accounting for the observed structural fluctuations during the simulation (Figure 8E). Solvent-accessible surface area (SASA), defined as the surface area of a protein molecule accessible to solvent molecules, was one of the key indicators for assessing protein folding and stability [38]. The SASA was slightly reduced in our simulations, which was beneficial for enhancing the stability of the proteins (Figure 8F). The radius of gyration (Rg), defined as the mass-weighted root-mean-square radius of the protein–ligand complex, was often used to assess conformational tightness and folding stability during binding simulations [39]. The lower the Rg value, the more compact the protein structure. Figure 8G demonstrated that the final Rg was slightly lower than the starting value, suggesting that the complexes are stable throughout the simulation. Additionally, the ligand consistently formed four hydrogen bonds with amino acids of the protein pocket throughout the 100 ns MD simulations, which significantly enhanced binding affinity (Figure 8H). The energy trap, revealed by energy analysis, was shown in Figure 8I. A free energy landscape is used to describe the free energy distribution of all conformations obtained from structural dynamics simulations of a complex, where the minimum energy corresponds to the most thermodynamically stable conformation. In Figure 8I, the blue color corresponds to the energy minimum, i.e., the most stable structure. The blue region slightly below the center of the diagram represents the lowest free energy area, indicating the preferred conformation of the molecule. The purer the blue, the lower the energy value and the more stable the structure. In contrast, the red region represents unstable structures.

## 4. Discussion

Spinosin, the primary active C-glycoside flavonoid constituent of *Semen Ziziphi Spinosae*, has garnered significant research interest due to its diverse pharmacological activities, notably its hypnotic and sedative effects [10,35,40]. Spinosin (10 and 15 mg/kg, p.o.) has been observed to significantly potentiate pentobarbital-induced sleep in mice assessed by the LORR, and at 15 mg/kg, it also reduces sleep latency and increases total sleep time in p-chlorophenylalanine (PCPA)-induced insomnia mice [36]. Wang et al. have revealed that spinosin (15 mg/kg, i.g.) significantly augmented pentobarbital-induced sleep in rats, with reduced sleep latency and increased total sleep time, NREM sleep time, and REM sleep time [41]. Our previous study, which assessed the hypnotic effects of spinosin (10, 20, 40 mg/kg) in male C57BL/6 mice via polysomnographic recordings, demonstrated that intraperitoneal injection of spinosin exerted dose-dependent hypnotic effects, with 40 mg/kg showing the strongest effect and significantly accelerating sleep onset [35]. Differences in spinosin dosage may arise from variations in sleep assessment methods, mouse strains, and administration routes. Building on our prior work, the present study showed that spinosin (40 mg/kg, i.p.) significantly shortened sleep latency, reduced wakefulness, and increased NREM sleep, thereby laying the foundation for subsequent mechanism research.

Quantitative analysis of sleep architecture employs EEG and EMG recordings to characterize the frequency of occurrence, the average duration, and state transitions of wakefulness, NREM, and REM sleep. The number of episodes of wakefulness, NREM sleep, and REM sleep is quantified as discrete events across the sleep period, reflecting sleep fragmentation and architecture [42]. Consistent with our previous reports [35], the present data showed that spinosin significantly increased the number of wakefulness episodes and NREM sleep episodes (Figure 3A), while it had a slight increase in the mean duration of NREM sleep (Figure 3B). Additionally, we newly found that spinosin at 40 mg/kg slightly increased the transition frequency between wakefulness and NREM sleep and the number of NREM sleep bouts (Figure 3C,D). The number of NREM sleep bouts refers to the count of continuous segments of NREM sleep throughout the night, serving as an important indicator for assessing sleep continuity and quality [43]. These results indicate that spinosin may improve sleep–wake behaviors by altering sleep architecture. NREM sleep is characterized by an EEG dominated by high-amplitude, low-frequency delta oscillations at frequencies of 0.5–4 Hz [44]; hence, delta power is a reliable indicator of sleep need during NREM sleep [45]. In the present study, compared to the control group, spinosin significantly increased the delta power density during NREM sleep in mice (Figure 3E), suggesting enhanced sleep depth [46]. The assessment of spontaneous locomotor activity serves as a primary pharmacological screening method for evaluating sedative-hypnotic agents [47]. In this study, the effect of spinosin on spontaneous locomotor activity was investigated using the OFT, and the data revealed that spinosin significantly decreased total moving distance in mice (Figure 2B,C). However, previous research showed that spinosin at a dose of 5 mg/kg (p.o. or i.g.) did not significantly decrease total distance traveled in anxiety-model mice [9,48]. Differences in outcomes may stem from the choice of normal mice as the model system and from the administered dose and route of spinosin. Collectively, these findings provide an additional scientific basis for spinosin’s sleep-promoting effects. However, research on the hypnotic mechanism of spinosin remains limited.

Emerging evidence suggests that A_2A_R plays a key role in adenosine-mediated hypnotic effects [49]. When delivered into the subarachnoid space beneath the rostral basal forebrain, CGS21680 (an A_2A_R-selective agonist) robustly enhances NREM sleep, in contrast to the minimal and variable effects observed with A_1_R agonists [50,51,52]. Caffeine promotes wakefulness primarily by blocking the A_2A_R [53,54]. In addition, Caffeine enhances locomotor activity by blocking the A_2A_R [55], and whereas spinosin effectively counteracts these hyperactivities induced by caffeine [56]. Therefore, the sleep-promoting effects of spinosin may be related to the A_2A_R, though further evidence is needed. Currently, caffeine has been used to investigate the role of A_2A_R in mediating hypnotic effects [57,58,59]. Pretreatment with caffeine (10 mg/kg, i.p.) completely antagonizes the hypnotic effects induced by ethanol (3 g/kg) [57] or myricitrin (10 mg/kg) [58] in mice. Kim et al. [59] demonstrates that the hypnotic effect of 3 mg/kg of luteolin is almost completely blocked by oral administration of caffeine pretreatment (10 mg/kg) in mice. In our preliminary dose-finding experiment, no antagonistic effect of caffeine (i.p.) against the sleep-promoting activity of spinosin (40 mg/kg, i.p.) was observed at the doses of 1 mg/kg and 3 mg/kg, while a significant antagonistic effect was detected at 5 mg/kg. The varying caffeine doses may stem from differences in subjects and administration methods, as indicated by these studies. In the present study, pretreatment with 5 mg/kg caffeine (i.p.) was sufficient to antagonize the hypnotic effects of spinosin, as evaluated by the PIST, EEG/EMG recordings, and OFT (Figure 1 and Figure 2). These data indicate a role for A_2A_R in mediating the hypnotic effects of spinosin. A_2A_Rs are enriched within the ventral striatum, with particularly high densities in the NAc subregion [60]. Local deletion of A_2A_R in NAc blocked caffeine-induced wakefulness [54]. Optogenetic or chemogenetic activation of NAc A_2A_R-expressing medium spiny neuron (MSN) robustly induced NREM sleep [61]. In summary, we propose that caffeine may block spinosin-induced adenosine binding to NAc A_2A_Rs.

However, our data showed that pretreatment with caffeine failed to significantly antagonize spinosin-induced changes in the episode number of each sleep–wake stage and the delta power density of NREM sleep (Figure 3). Several studies have reported the sleep-promoting mechanisms of spinosin. In 2008, Wang et al. [36] found that 5-HT (5-hydroxytryptamine) receptor subtypes may be involved in the hypnotic effects of spinosin. Further studies reported that spinosin could reverse the 8-OH-DPAT (a 5-HT_1_A receptor agonist)-induced reductions in NREM sleep, REM sleep, and SWS time in pentobarbital-treated rats, which suggests that spinosin may be an antagonist at postsynaptic 5-HT_1_A receptors [41]. Wang et al. [62] also showed that presynaptic 5-HT_1_A autoreceptor mechanisms may be involved in the potentiating effect of spinosin on pentobarbital-induced LORR in mice. In addtion, spinosin may promote NREM sleep and enhance sleep quality by regulating neuronal activity in key brain regions, especially by GABAergic neurotransmission and orexinergic systems [35,63]. Dong et al. [64] also showed that a modified Ziziphi Spinosae Decoction enriched with spinosin improved sleep in insomnia mouse models, likely through modulation of the orexin system. Hence, spinosin exerts hypnotic effects by regulating the neurotransmitter system through multiple targets and pathways. Similar results have been reported, demonstrating that caffeine antagonism of A_2A_R did not reverse the increased delta power density induced by ethanol [57]. However, how spinosin affects these sleep architecture indicators and delta power density of NREM sleep requires further study. Collectively, our data support a role for A_2A_R in mediating the hypnotic effects of spinosin.

Constitutive genetic knockout generates mouse models with complete loss of function in target receptors, thereby enhancing the precision of studies on the roles and mechanisms of brain receptors. To further validate the hypnotic mechanism of spinosin via the A_2A_R, we conducted parallel experiments in A_2A_R-KO mice and their A_2A_R-WT littermates. Consistent with caffeine pretreatment, EEG/EMG recordings showed that compared to their A_2A_R-WT littermates, the hypnotic effects of spinosin were significantly reduced in the A_2A_R-KO mice (Figure 4). Spinosin and myricitrin are both types of flavonoids. Kim et al. [58] found that the hypnotic effects of myricitrin were mediated by A_2A_R. Similarly, in the OFT, the reducing effect of spinosin on the locomotor activities was not significant in the A_2A_R-KO mice (Figure 5). Hole-cross test has demonstrated that caffeine significantly increased the crossing frequency and locomotor activity in mice, whereas spinosin effectively counteracted these hyperactivities induced by caffeine [56]. Chiu et al. [65] reported that administration of adenosine in WT mice led to a 33% reduction in spontaneous locomotor activity, which was not observed in A_2A_R-KO mice. Taken together, these findings strongly suggest that the A_2A_R mediates the hypnotic effects of spinosin.

The regulation of sleep and arousal depends on the coordinated activity of wake-promoting and sleep-promoting neurons, which mutually inhibit and compete for network dominance, creating a systematic “switch” that results in either the sleep or awake state [66,67]. Our previous study has revealed that spinosin (40 mg/kg, i.p.) significantly enhanced c-Fos and DARPP-32 co-expression in the NAc and reduced c-Fos and orexin-A co-expression in the LH, but had no significant effect on c-Fos co-expression with TH (tyrosine hydroxylase) in the ventrolateral periaqueductal gray (VLPAG) or with 5-HT in the dorsal raphe (DR) [35]. Therefore, we evaluated the effects of caffeine pretreatment or A_2A_R deletion on spinosin-induced changes in the activity of NAc GABAergic MSNs and LH orexin neurons, using c-Fos immunostaining. In this study, we observed that spinosin’s effect on the activity in NAc GABAergic MSNs and LH orexin neurons was significantly reversed by caffeine pretreatment or by A_2A_R deletion (Figure 6 and Figure 7). A_2A_Rs are abundantly expressed on the NAc GABAergic MSNs [24,25]. Optogenetic and chemogenetic activation of NAc A_2A_R neurons promotes NREM sleep, whereas NAc A_2A_R neuronal inhibition suppresses NREM sleep [61]. Adenosine, acting through NAc A_2A_R, induces NREM sleep [68]. Caffeine promotes wakefulness by antagonizing the A_2A_Rs in NAc [54]. Thus, spinosin likely promotes sleep via A_2A_R-mediated activation of the NAc GABAergic MSNs. Orexin neurons, primarily located in the LH, are known to be implicated in sleep–wakefulness regulation, particularly the maintenance of wakefulness [69]. Optogenetic and chemogenetic activation of LH orexin neurons induces and maintains wakefulness, whereas inhibition promotes NREMS [70,71,72,73]. Hence, we propose that the hypnotic effects of spinosin may also be related to the inhibition of the LH orexin neurons.

Currently, molecular docking has revealed that caffeine and adenosine bind with high affinity to ADORA2A [74]. The present study revealed that the binding energy of spinosin and ADORA2A was −7.9666 kcal/mol, which was more negative than that of caffeine-ADORA2A (−6.0082 kcal/mol) and adenosine-ADORA2A (−6.6236 kcal/mol) (Figure 8A–C). In molecular docking, a binding energy below −5.0 kcal/mol is generally considered indicative of good affinity, while values below −7.0 kcal/mol suggest strong affinity. Lower docking energies correlate with stronger ligand–protein interactions [75]. In addition, spinosin formed more hydrogen bonds with ADORA2A than either caffeine or adenosine did. These results suggest that spinosin could bind to ADORA2A with high binding affinity.

Despite its effectiveness in predicting protein–compound interactions, molecular docking’s accuracy is constrained by factors such as protein flexibility and solvation effects. MD simulations, widely used to model the dynamic behavior of flexible proteins and ligands over time, reveal changes in stability, structural conformation, binding states, and have become a validated tool for assessing molecular docking outcomes [76]. In this study, MD simulations further demonstrated that spinosyn–ADORA2A complexes remained stable under simulated physiological conditions (Figure 8D–I), indicating that the binding could be maintained in vivo.

To date, no human clinical trials on pure spinosin have been published, but a number of clinical trials have verified that Chinese herbal formulae containing *Semen Ziziphi Spinosae* (with spinosin as the core active component) can significantly improve sleep quality in patients with primary insomnia, and cause fewer side effects than Diazepam [77]. The pharmacokinetic study showed that spinosin quickly accumulates in the brain first, and the sedative and hypnotic effects of spinosin are closely related to its concentration in the brain after intravenous administration to rats (3.49 and 5.93 μg/g at 5 min and 20 min, respectively) [78]. An early study reported that no adverse effects were observed in mice even at the highest dose of spinosin (10 g/kg, i.p.) [56]. Zhang et al. [63] significantly enhanced the solubility and oral bioavailability of spinosin through solid dispersion technology and incorporated it into functional milk beverages to improve sleep disorders. Hence, spinosin, a natural compound with low toxicity, good bioavailability, and multi-target effects, significantly increases delta power, thereby providing a theoretical rationale for further investigation into its potential as a candidate for novel non-benzodiazepine sleep aid development. Notwithstanding, further investigation is warranted to bridge the translational gap between preclinical efficacy and real-world deployment, with comprehensive pre-commercial assessments of stability, bioavailability and human safety required.

Despite these promising findings, there are some limitations in our study.The study has a relatively small sample size (*n* = 5 per group), which reduces statistical power. The exclusive inclusion of male mice precludes extrapolation of results to female subjects. We also clarified that although no severe adverse reactions were observed, a formal acute toxicity test for the selected spinosin dose (40 mg/kg, i.p.) was not conducted in this study. Furthermore, the direct binding interaction between spinosin and A_2A_R requires further verification through targeted biophysical assays, such as surface plasmon resonance (SPR). Finally, due to time and resource limitations, we were unable to experimentally confirm all of the expected targets outside of A_2A_R. We will conduct a large-sample study in male and female mice to elucidate the molecular pathways underlying the A_2A_R-mediated hypnotic effect of spinosin and to systematically evaluate its preclinical safety profile.

## 5. Conclusions

In conclusion, our study characterized the hypnotic profile of spinosin in mice using PIST, OFT, and EEG/EMG recordings, and demonstrated that it significantly increases the transition frequency between wakefulness and NREM sleep, the number of NREM sleep bouts lasting 32–64 s, and the delta power density during NREM sleep. The hypnotic effects of spinosin and its modulation of the activity of NAc GABAergic MSNs and LH orexin neurons were significantly reversed in caffeine-pretreated or A_2A_R-KO mice, indicating spinosin-induced hypnosis is mediated by the A_2A_R. Moreover, molecular docking and MD simulations confirmed that spinosin stably bound to the A_2A_R. Taken together, we propose spinosin as a promising candidate for the treatment of insomnia.

## Data Availability

The original contributions presented in this study are included in the article. Further inquiries can be directed to the corresponding author.

## Supplementary Materials

The following supporting information can be downloaded at: https://www.mdpi.com/article/10.3390/nu18111785/s1, Figure S1: Structure of spinosin.
