# Supplementary figures and images for: The Hypnotic Effect of Spinosin Is Mediated by Adenosine A2A Receptors in Male Mice
Source: Nutrients. 2026 Jun 1;18(11):1785. doi: 10.3390/nu18111785 (PMC13258738; doi:10.3390/nu18111785)

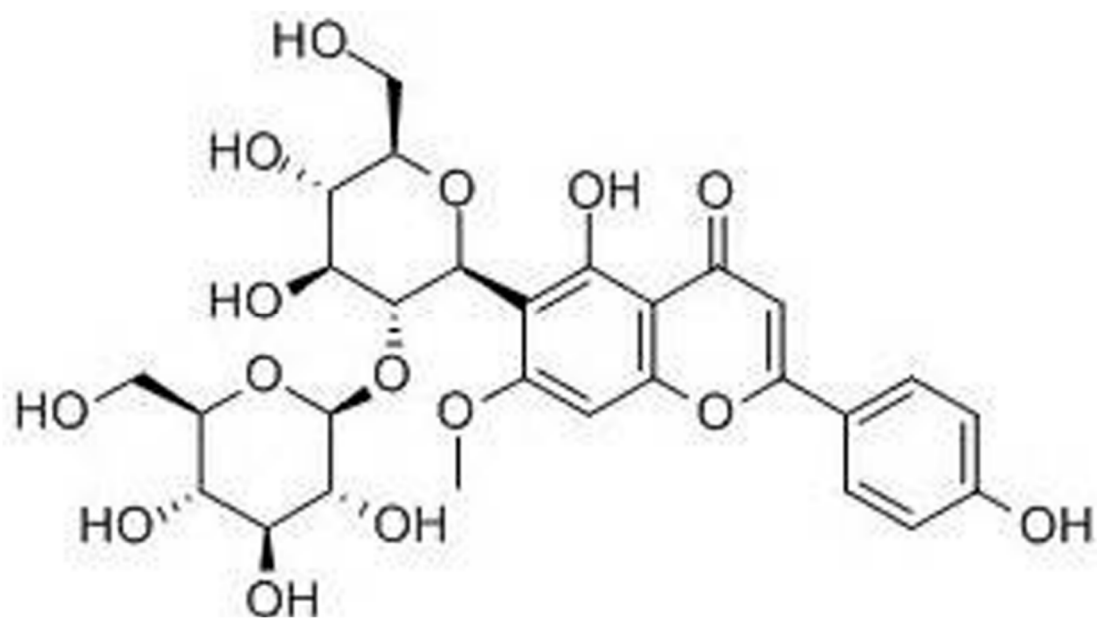

Figure S1. Structure of Spinosin

Supplement: Supplementary file 1 [file nutrients-18-01785-s001.zip › nutrients-4304375-supplementary.pdf]
